# Intelligence

**Published:** 1832-06

**Authors:** 


### INTELLIGENCE.

### UNIVERSITY OF LONDON.

On Wednesday, the 15th of May, the Prizes in the Medical School of this Institution were delivered. The theatre was crowded with strangers and practitioners of medicine; Dr. Lushington was in the chair. After a few prefatory remarks from the chairman, a Report to the Council was read by Dr. A. T. Thomson of the state of the School. Two hundred and fifty students have this session entered the medical classes.

The following are the names of the gentlemen who obtained medical prizes.

Materia Medica, (Professor Dr. A. T. Thomson.) Gold medal, Francis Taylor, of York; first silver ditto, Michael Foster, Bedfordshire; second silver ditto, George Allarton, Warwickshire.

Anatomy, (Professor Dr. Quain.) Gold medal, George Houlton, Lincolnshire; first silver ditto, Charles Nossoc, London; second silver ditto, William Rayner, Lincolnshire.

Practical Anatomy, (Demonstrator, Mr. R. Quain.) Gold medal, William Rayner, Lincolnshire; first silver ditto, W. K. Wright, Bristol; second silver ditto, John Bartlett, Great Bedwin.

Practice of Medicine, (Professor Dr. Elliotson.) Gold medal, James Wearne, St. Ives; first silver ditto, John Storrar, London; second ditto, J. P. Wall.

Surgery, (Professor Samuel Cooper, Esq.) Gold medal, R. Lanyon, Cornwall; first silver ditto, William Rayner, Lincolnshire; second ditto, David Hartley, Bristol.

Midwifery, (Professor Dr. D. D. Davis.) Gold medal, G. P. Gill, London; first silver ditto, Wm. Rayner, Lincolnshire; second ditto, J. N. Huddlestone, London.

Chemistry, (Professor Dr. Turner.) Gold medal, D. B. Meek: first silver ditto, H. Cook, Hampstead; second ditto, A. M. A'Beckett, London.

Comparative Anatomy, (Professor Dr. Grant.) Gold medal, D. W. Nash, Bristol: silver ditto, John Storrar, London.

Botany, (Professor John Lindley, Esq.) Gold medal, R. B. Hinds; silver ditto, J. H. Rogers.

Medical Jurisprudence, (Professor Dr. A. T. Thomson.) The prize, P. H. Chavasse, of Birmingham.

In concluding the business of the day, the chairman delivered an eloquent and impressive address to the students.

### KING'S COLLEGE, LONDON.

*Prizes awarded, and publicly presented to the Students of the Medical School, on Friday,* 18*th May,* 1832.

His Grace the Bishop of Llandaff in the chair; present, many members of the Council, the Professors, and a very numerous assemblage of noblemen and gentlemen, friends of the Institution.

The ceremony commenced by the very Reverend Prelate who presided stating, in a neat and appropriate address, the objects for which the meeting had been convened, viz. the public distribution of rewards to those medical students who had honourably distinguished themselves during the session just concluded; rewards which were designed not only to express the approbation of the Council towards them for their proficiency, but also to act as further excitements to their fellow-students who had been less successful than themselves, but who appeared, from the reports of the Principal and Professors, not to have failed from any absolute deficiency, but only from the superior attainments of the present prizemen, and who would certainly have received rewards, as they deserved them, only that in such cases few only can win, though many may compete; and hence he was anxious to congratulate even those whose names would not be mentioned, upon the very creditable examinations they had passed, of which the reports made by the several Professors were most satisfactory and favorable, and to assure them that, in such competitions, even to have tried is honourable, and that defeat is not disgrace.

The Reverend the Principal then announced the mottoes of the successful candidates, which, when opened by the Bishop, were found to belong to the following gentlemen, who were introduced to the chairman by the several Professors, and received, along with their prizes, commendations from him, and hearty commendations from their fellow-students and the company assembled. We subjoin a list of the successful candidates in the various classes, and copies of two or three of the examination papers.

At the close of the distribution the President again addressed the meeting, and especially the students, expressing the sincere gratification he felt, (and which appeared to be participated in by all present,) at the ceremony they had witnessed, and in which the latter indeed had been the principal actors, and which, as the first-fruits of the College system of education, gave promise of a most abundant harvest.

The distinguished Prelate concluded by informing the meeting, that the distribution of prizes in the classical department would take place in the first week in July.

*Gentlemen who received Prizes.*

In Anatomy, to Mr. Mark Noble Bower, from Birmingham.

Anatomical Demonstrations: First prize, Mr. M. N. Bower; second ditto, Mr. John Soden, from Bath.

Botany: First prize, Mr. Philip King Weston, London; second ditto, Mr. Wm. Benson Whitfield, London; third ditto, Mr. Charles Roper, Hackney.

Chemistry: First prize, Mr. Wm. Thomas Christopher Robinson, London; second ditto, Mr. A. H. Talmadge, from Durham.

Surgery: First prize, Mr. Charles Carter, from Newcastle upon Tyne; second ditto, Mr. John Tomkins, London.

Materia Medica: First prize, Mr. Edward John Chance, London; second ditto, Mr. Henry Curling, London.

Medicine: First prize, Mr. E. J. Chance; second ditto, Mr. W. B. Whitfield.

Midwifery: Mr. E. J. Chance.

*Questions in Anatomy.*

1. Describe the mechanical structure of Bone.
2. What is an Epiphysis?
3. Enumerate the Cartilages of the Larynx, and describe the nature of the Ligamenta Glottidis.
4. Enumerate the Diseases to which the bones are liable.
5. Enumerate the Muscles which bend the Knee-joint.
6. Enumerate the Muscles of Inspiration.
7. Describe the Ligaments of the Shoulder-joint, and the different ways in which the head of the Humerus may be dislocated.
8. Describe the distribution of the second division of the Fifth Nerve.
9. Enumerate the Ligaments of the Knee-joint.
10. Describe the Fœtal Circulation.
11. What is the origin of the Phrenic Nerve?
12. What are the Nerves which have Ganglions?

13. Describe the course, and name the branches, of the Subclavian Artery.

14. Describe the relative situations of the Artery and Vein in the following instances: common Carotid, Subclavian, Axillary, external Iliac, common and superficial Femorab.

*Questions in Midwifery.*

1. Describe the Female Pelvis, and state the dimensions of the various diameters of its upper aperture, its cavity, and of its lower aperture.

2. What is meant by the Axis of the Pelvis ?

3. Describe the Female Organs of Generation in the adult unimpregnated state.

4. What changes do these organs undergo during pregnancy ?

5. Required the descriptive Anatomy of the Ovum, viz. the enumeration and relative situation of its various parts, their form, structure, and supposed uses.

6. What are the signs of Pregnancy; with what diseases may pregnancy be confounded; and by what means can it be detected ?

7. What is meant by Extra-Uterine Pregnancy? How many kinds are there?

8. What changes are produced in the mother, and in the fœtus in extra-uterine fœtation ?

9. How is the Fœtus nourished in Utero ?

10. Describe the Fœtal Circulation.

11. By what powers is the child expelled?

12. Describe the Position of the Fœtus in the various stages of natural labour.

13. How are the Uterine Pains produced? By what laws are they governed; what are their deviations; and how may these be remedied?

14. How may "Unnatural Labours" be conveniently classified ?

15. What are the most frequent of the Unnatural Presentations of the Fœtus? and how are they distinguished?

16. How many kinds of Uterine Hæmorrhage are there; what are the symptoms of uterine hæmorrhage?

17. What is the treatment of Uterine Hæmorrhage, when it occurs in the early months of pregnancy. 2. In the latter months. 3. During labour. 4. After labour.

18. What are the signs by which the Placenta is known to present; and what is the practice in such cases?

19. What is the order of symptoms in Convulsion?

20. In what cases is Turning necessary? State the precautions necessary to be taken in turning, and describe the operation.

21. What is meant by Spontaneous Evolution? Describe its mechanism.

22. What are the general rules for delivery with the Short Forceps?

23. What are the circumstances which render Craniotomy necessary? Describe the operation.

24. What are the means proposed to prevent the necessity of Craniotomy; and what is the efficacy of these means?

*Questions in Chemistry.*

1. Whence do we derive our first idea of Force ? And how do we roughly estimate different degrees of Mechanical Forces?

2. Distinguish some of the principal Attractive Forces which act upon matter.

3. Distinguish some of the principal Repulsive Forces.

4. Whence do we derive our first notion of Heat?

5. What is the most universal effect of Heat?

6. Explain the construction of Thermometers, and the mode by which observations with them are rendered comparable in all places and ages.

7. Upon what is the graduation of Fahrenheit's scale founded?

8. When the Equilibrium of Temperature has been disturbed, how is it restored in Solids, Liquids, and Aëriform Fluids? And how between bodies not in contact?

9. What is the Law of Expansion by Heat in aëriform fluids?

10. If two equal measures of the same fluid be mixed together, of different temperatures, what will be the temperature of the mixture as indicated by the thermometer?

11. If two equal measures of dissimilar fluids, such as water and oil, of different temperatures, be mixed together, will a similar result be obtained? If not, state upon what principle the difference is founded.

12. What phenomena of heat are observable, when a body changes its physical state?

13. State the difference between the Attraction of Cohesion, and Chemical Affinity, and give an example of any influence which the former may exercise upon the latter.

14. How many degrees of Chemical Affinity may you distinguish? Particularize the limits of combination under each degree, giving examples of each.

15. When a body combines with another body in more than one proportion, is there any general law by which such combination is regulated? Give examples, with reference both to weight and volume.

16. What is meant by the term Chemical Equivalents? Give an illustration of the meaning.

17. Give examples of what is called Single Elective Affinity, and of Double Elective Affinity.

18. Have the properties of Solubility and Elasticity any influence upon Elective Affinity? If any, give examples of their influence.

19. What is meant by Chemical Elements and Proximate Principles?

20. When a chemical compound is subjected to the action of the Voltaic Pile, what is the result?

21. Describe the phenomena when Sulphate of Potassa, Sulphuric Acid, and Potassa are severally subjected to its action.

22. Enumerate some of the most Electro-Negative, and some of the most Electro-Positive simple bodies, and state their equivalent numbers with reference to Hydrogen.

23. Is the distinction of Electro-Positive and Electro-Negative, absolute or comparative?

24. Mention the names of some of the principal Hydro-Acids with their equivalent numbers, and explain any peculiarity which attends their combination with Metallic Oxides.

25. When Muriatic Acid is made to act upon Soda, what is the result? and what explanation may be given of the action?

26. Describe the best process for the detection of Arsenic.

27. What kind of Precipitate does Arsenious Acid occasion with Nitrate of Silver? and how may it be distinguished from that occasioned by Phosphoric Acid?

28. By what test may the Arsenious be distinguished from the Arsenic Acid?

29. By what tests may you distinguish Potassa from Soda in solution, Baryta from Strontia, and Magnesia from the other Earths?

30. Describe the best process for the ultimate Analysis of Organic Compounds, and explain the principle upon which it is founded.

---

*Guaco Juice.* THE Medico-Botanical Society has just received a quantity of the juice of the Mikania Guaco, the plant which is said to be an antidote to the poison of the rattle snake, and which has been recommended for the treatment of hydrophobia; and Earl STANHOPE, the president of the Society, announced, at the last meeting, that any medical practitioners might be supplied with the juice on application to the secretaries, on the very liberal conditions that the results of their experiments should be communicated to the Society, for the benefit of the public at large.

---

*Death of a Gentleman from Carelessness in dispensing Medicine.*

A FEW days ago, a verdict of manslaughter was returned against the assistant of a chemist at Brighton, for making a mistake in dispensing a prescription, by which Captain BURDETT, of the Royal Navy, lost his life. It appears that oil of tar was substituted for infusion of senna. Nothing can be further from our intention in mentioning this lamentable accident than to add to the mental distress which is doubtless felt by the person who was the cause of it; but we must add, that, in our opinion, the law does well to shew that greater care and attention are expected from young men to whom the highly responsible duty of dispensing medicines is extended. We wish also it were possible to inflict some punishment upon all medical practitioners who write their prescriptions in an almost illegible hand. We have seen many prescriptions in which it was difficult to make out the remedy, while the doses could only be guessed at; and we once remember that upon one of these disgraceful specimens being submitted to the writer for explanation, *he could not read it himself.* If, in such a case, any mistake should happen in dispensing the medicine, the writer of the prescription is morally and ought to be considered legally, a *particeps criminis.*

---

### OBITUARY.

MAY 17.

Walter R. H. Barker, no address
Henry Franklin Foley, of Durham
Edward Gee, Bristol
William Harding, Ludlow
Wm. Bakewell Matthews, Ashby de la Zouch
Wm. Henry Power, no address
George Miller Prichett, Yeovil
Edward Tippetts, Dartford

MAY 24.

Arthur Abraham Brett, of Wisbeach
Augustus Frederick Chalk, Dover
William Case, Portsmouth
Issaac Flower (of Knook, Wilts.), London
George Horton, Bromsgrove
Thomas Pedder Hutton, Beetham
Robert Hope Jones, Conway
William Jawell Roper, Norwich
Thomas Sadler, Cheltenham
Thomas Tweddle, Longtown
Charles Westron, Wellington
William Wheeler, Leominster
Joseph Walker, Belper.

### METEOROLOGICAL JOURNAL,

By Messrs. HARRIS and Co. Mathematical Instrument Makers, 50, High Holborn.

| | Moon. | Rain gauge. | Thermometer. | | | Barometer. | | De Luc's Hygrometer | | Winds. | | Atmospheric Variation. | | |
|---|---|---|---|---|---|---|---|---|---|---|---|---|---|---|
| April | | | 9a.m. | max. | min. | 9a.m. | 10p.m. | 9a.m. | 10p.m. | 9 a.m. | 10p.m. | 9 a.m. | 2 p.m. | 10 p.m. |
| 20 | | | 50 | 52 | 41 | 29.46 | 29.64 | 72 | 74 | SW v. | SW | showery | rain | fine |
| 21 | | .18 | 47 | 53 | 43 | .90 | .90 | 75 | 75 | W | SW | fine | fine | |
| 22 | | | 49 | 61 | 45 | .76 | .82 | 63 | 55 | SW | S | | | |
| 23 | ☾ | | 53 | 59 | 39 | .68 | .52 | 49 | 53 | S | SSW | | | |
| 24 | | | 42 | 50 | 44 | .53 | .61 | 54 | 54 | N | NNW | | showery | rain |
| 25 | | .25 | 51 | 55 | 45 | .68 | .60 | 54 | 57 | NW | WSW | cloudy | rain | fine |
| 26 | | | 49 | 54 | 42 | .52 | .63 | 57 | 54 | SSE | NE | | | cloudy |
| 27 | | .09 | 50 | 52 | 38 | .65 | .67 | 53 | 54 | E | ENE | fine | fine | fine |
| 28 | | | 49 | 49 | 42 | .54 | .44 | 54 | 50 | ENE | ESE | cloudy | | |
| 29 | | | 51 | 55 | 40 | .27 | .15 | 50 | 50 | NE | NE | fine | | |
| 30 | ● | | 54 | 56 | 43 | .11 | .29 | 51 | 54 | ENE v. | NE | cloudy | | |
| May 1 | | | 47 | 57 | 47 | .22 | .15 | 59 | 60 | SE | WSW | rain | rain | |
| 2 | | .46 | 57 | 58 | 50 | .15 | .19 | 60 | 61 | SSW | SW v. | | cloudy | cloudy |
| 3 | | | 55 | 56 | 47 | .25 | .13 | 58 | 60 | SW | SW | cloudy | rain | fine |
| 4 | | .15 | 49 | 54 | 45 | .51 | .81 | 60 | 58 | E | SE | | fine | |
| 5 | | | 51 | 57 | 48 | .79 | .83 | 64 | 64 | WSW | SW | | showery | rain |
| 6 | | | 55 | 63 | 55 | .75 | .69 | 65 | 66 | SW | SW | | fine | fine |
| 7 | ☽ | .31 | 62 | 66 | 51 | .67 | .69 | 66 | 60 | ESE | NW | fine | | |
| 8 | | | 62 | 64 | 41 | .82 | .92 | 58 | 52 | W | N | | | |
| 9 | | | 51 | 54 | 40 | 30.07 | 30.13 | 48 | 47 | ENE | NE | | | |
| 10 | | | 50 | 53 | 42 | .16 | .19 | 47 | 48 | NE v. | NE v. | | | |
| 11 | | | 52 | 54 | 41 | .11 | 29.93 | 50 | 48 | NNW | N | | | rain |
| 12 | | | 44 | 47 | 43 | 29.74 | .58 | 48 | 47 | N | NW | | | fine |
| 13 | | | 46 | 52 | 40 | .65 | .61 | 49 | 51 | NW | NW | | cloudy | |
| 14 | | | 50 | 54 | 38 | .58 | .55 | 51 | 53 | NNW | ENE | | fine | cloudy |
| 15 | ○ | | 43 | 52 | 39 | .57 | .62 | 53 | 54 | E | ENE | cloudy | rain | cloudy |
| 16 | | | 47 | 56 | 43 | .63 | .68 | 54 | 60 | ENE | ESE | | fine | fine |
| 17 | | .12 | 50 | 60 | 45 | .63 | .75 | 59 | 58 | N | E | | cloudy | cloudy |
| 18 | | .20 | 55 | 60 | 46 | .87 | .95 | 56 | 54 | E | SE | fine | fine | fine |
| 19 | | | 57 | 61 | 46 | 30.01 | 30.00 | 50 | 45 | SSE | S | | | |

The quantity of Rain fallen in the month of April, was 64.100ths of an inch.

